# Prevalence and Risk Factors Associated with Dyslipidemia in Chongqing, China

**DOI:** 10.3390/ijerph121013455

**Published:** 2015-10-26

**Authors:** Li Qi, Xianbin Ding, Wenge Tang, Qin Li, Deqiang Mao, Yulin Wang

**Affiliations:** 1 Chongqing Municipal Center for Disease Control and Prevention, Chongqing 400042, China; E-Mails: qili19812012@126.com (L.Q.); dingxianbing1970@163.com (X.D.); wengetang@163.com (W.T.); cqcdcliqin@126.com (Q.L.); molly19812010@163.com (D.M.); 2 Department of Epidemiology, College of Prevention Medicine, the 3rd Military Medical University, Chongqing 400038, China

**Keywords:** dyslipidemia, prevalence, risk factors

## Abstract

The increasing prevalence of dyslipidemia has become a worldwide public health problem, and the prevalence varies widely according to socioeconomic, cultural and ethnic characteristics. Chongqing has experienced rapid economic development and is now the economic center of Southwestern China. There are scant data on serum lipid profile of residents in Chongqing, the largest municipality directly under the Central Government in China. We conducted a cross-sectional study in a representative sample of 5375 residents of Chongqing, aged ≥18 years, and estimated the prevalence of dyslipidemia and its associated risk factors. According to the National Cholesterol Education Program-Adult Treatment Panel III criteria, the age-standardized prevalence of dyslipidemia was 35.5% (34.4% among men and 37.6% among women). Among the 2009 patients with dyslipidemia, 44.2% had isolated hypertriglyceridemia, 14.7% had isolated hypercholesterolemia, 13.2% had mixed hyperlipidemia, and 28.0% had isolated low high-density lipoprotein cholesterol. The peak prevalence of dyslipidemia in men was between 30 and 39 years (48.2%), and then declined gradually; in women, the prevalence of dyslipidemia increased with age, with the peak prevalence occurring after age 60 (46.3%). Multivariable logistic regression analysis revealed that dyslipidemia was associated with age, education level, physical activity, obesity and central obesity for both men and women. In conclusion, the results indicated dyslipidemia, particularly hypertriglyceridemia and low high-density lipoprotein cholesterol, are very common in Chongqing. To prevent dyslipidemia, it is essential to conduct appropriate intervention programs aimed at risk factor reduction and implement routine screening programs for blood lipid levels in Chongqing, China.

## 1. Introduction

Dyslipidemia is one of the major modifiable factors for the development of type 2 diabetes [1,2], atherosclerosis [3,4], stroke [5,6] and cardiovascular diseases [7,8]. With rapid socioeconomic development and associated lifestyle changes, the prevalence of dylipidemia has increased dramatically over the past decade in China [9,10,11]. 

As the largest municipality of China, Chongqing has experienced rapid economic development and has become the economic center of Southwestern China. At the same time, its residents have experienced huge lifestyle changes, such as Westernization of diet, reduced physical activity and long-term sedentary work, all of which are regarded as major risk factors for dyslipidemia. Precise estimation of the frequency and patterns of dyslipidemia is essential for proper planning of health actions for prevention of negative clinical consequences. However, to the best of our knowledge, until now, no study has been conducted to allow for estimation of the prevalence of dyslipidemia on population level in Chongqing. To address this gap in knowledge, we measured serum triglyceride, total cholesterol and high-density lipoprotein cholesterol in a representative sample of 5375 residents of Chongqing, aged ≥18 years, and estimated the prevalence of dyslipidemia for the overall population and subgroups according to gender, and explore its associated risk factors. We aimed at compiling useful information on the health profile of residents and create a database that can be useful to the local health professionals charged with controlling and managing dyslipidemia in Chongqing, China.

## 2. Materials and Methods

### 2.1. Population Sample

In the period February through April 2014, a cross-sectional field survey targeted adults, aged ≥18 years, was conducted by Chongqing Center for Disease Control and Prevention. Only persons who had been living in their current residence for at least 6 months were eligible to participate. Mentally or physically handicapped people and pregnant females were disqualified from the survey.

A multistage sampling method was used: Stage 1, nine regions were randomly selected from the whole of Chongqing. Stage 2, four towns were randomly selected from each of the sampled region. Stage 3, three administrative villages or communities were randomly selected from each of the sampled towns. Stage 4, households within each village or community were listed, and 50 households were randomly selected. In Stage 5, one adult, aged ≥18 years, was selected randomly from each household using a Kish selection table [12]. When the selected individual declined or was unavailable on three occasions, a replacement household was randomly selected from all households in the same community or village after excluding the already selected households.

Overall, a total of 5400 people were sampled and requested to participate in the field survey, 5375 of these people completed the survey and were included in the final analysis. The study protocol was approved by the ethical review committee of the Chongqing Center for Disease Control and Prevention (CDC). Written informed consent was obtained from all study participants.

### 2.2. Data Collection and Measurements

All interviewers attended a two-day intensive training session, before carrying out the survey. Materials containing information about the purpose of this study, the standard method of measurement, the proper administration of the questionnaire, the importance of standardization and the study procedures were prepared for every investigator.

Selected individuals were interviewed face-to-face by trained interviewers, with a questionnaire that inquired about demographic characteristics and health-related habits. 

Height and weight were measured twice using a height-weight scale that had been calibrated before Subjects stood with bare feet and wore light clothing, and then the averages were calculated. Body mass index (BMI) was calculated as weight (kg) divided by height (m) squared. Waist circumference (WC) was measured twice on standing participants at the midpoint between the lower edge of the costal arch and the upper edge of the iliac crest, and means were calculated. 

Venous blood samples were collected and centrifuged immediately for all participants in the morning after at least a 12-hour fast. Specimens were then frozen and stored at −70 °C within 2 h of collection, and serum lipid profiles, including total cholesterol, high-density lipoprotein cholesterol (HDL_C), and triglycerides were assayed by the automated spectrophotometer and enzymatic colorimetric method with the use of Olympus AU640 autoanalyzer (Olym-pus, Kobe, Japan). All control values were consistent with the standards recommended by the medical laboratory of China Center for Disease Control and Prevention.

### 2.3. Definitions of Variables

Dyslipidemia was diagnosed according to the criteria set by the National Cholesterol Education Program-Adult Treatment Panel III (NCEP-ATP III) and classified into four phenotypes [13]: (a) isolated hypertriglyceridemia was defined as having serum triglycerides ≥1.7mmol/L or on medication and total cholesterol <6.2 mmol/L; (b) isolated hypercholesterolemia was defined as having total cholesterol ≥6.2 mmol/l or on medication and triglycerides <1.7mmol/L; (c) mixed hyperlipidemia was defined as having triglycerides ≥1.7 mmol/L and total cholesterol ≥6.2 mmol/L; and (d) isolated low HDL-C was defined as having HDL-C ≤1.03 mmol/L in male and ≤1.29 mmol/L in female without hypercholesterolemia nor hypertriglyceridemia [14]. According to World Health Organization guideline [15,16], overweight was defined as a BMI ≥25.0 kg/m^2^ and <30.0 kg/m^2^, and obesity was defined as a BMI ≥30 kg/m^2^. Central obesity was defined as waist circumference ≥90 cm in men and ≥80 in women. Regular physical activity was defined as participation in moderate or vigorous activity for ≥30 minutes/day at least 5 days per week. Smoking was classified in terms of current smokers or non-smokers (including ex-smokers). Habitual alcohol consumption was defined as drinking twice per month over the past 12 months, regardless of the quantity of alcohol consumed [17]. 

### 2.4. Statistical Analysis

The mean and standard deviation were provided for continuous variables and the prevalence and corresponding 95% confidence intervals (CIs) were calculated for categorical variables. A Student *t*-test was used to test differences in the means of continuous variables, and the Chi-square test to test differences in categorical variables. The analyses were gender-specific. A multivariable logistic regression analysis, using a backward elimination method, was used to analyze the risk factors. A two tailed *p* value < 0.05 was considered statistically significant. All initial data was entered into Epidata software 3.1 versions and Statistical Package for Social Sciences 18.0 for statistical analyses.

## 3. Results 

### 3.1. Characteristics of Study Population

Among the 5400 initial participants in our study, 25 were excluded because we did not have complete data for them. A total of 5375 participants (2030 men and 3345 women), aged ≥18 years, were included in this study. Their mean age (SE) was 57.7 (13.1) years, which was higher for men than for women (59.7 years *vs.* 56.5 years, *p* < 0.05). Only 11.4% of the subjects had more than a junior high school education. The BMI of men and women were 23.4 ± 3.2 and 24.2 ± 3.4 kg/m^2^, respectively. The waist circumference of men and women were 82.6 ± 9.5 and 81.5 ± 9.5 kg/m^2^, respectively. The prevalences of overweight, obesity and central obesity were 29.3% (95% CI: 28.7%, 29.9%), 4.6% (95% CI: 4.3%, 4.9%) and 4.4% (95% CI: 4.3%, 4.4%), respectively, all of which were significantly higher in women than in men (*p* < 0.05).

### 3.2. Prevalence of Dyslipidemia

Table 1 presents the lipid level and prevalence of dyslipidemia in total and by different gender. Of the total 5375 subjects, 37.4% (95% CI: 36.7%, 38.0%) had dyslipidemia. After standardization of age, based on China’s 2010 census data, the prevalence was 35.5% (95% CI: 34.8%, 36.1%): 34.4% (95% CI: 33.3%, 35.4%) for men and 37.6% (95% CI: 36.7%, 38.4%) for women. 

Among the 2009 patients with dyslipidemia, 44.2% had isolated hypertriglyceridemia, 14.7% had isolated hypercholesterolemia, 13.2% had mixed hyperlipidemia, and 28.0% had isolated low HDL-C. Therefore, the prevalences of isolated hypertriglyceridemia, isolated hypercholesterolemia, mixed hyperlipidemia and isolated low HDL-C were 16.5% (95% CI: 16.0%, 17.0%), 5.5% (95% CI: 5.2%, 5.8%), 4.9% (95% CI: 4.6%, 5.2%) and 10.5% (95% CI: 10.0%, 10.9%) among the 5375 subjects, respectively. In addition, many of 692 participants with low HDL-C had other types of hyperlipidemia: 576 had isolated hypertriglyceridemia, 14 had isolated hypercholesterolemia and 102 had mixed hyperlipidemia. Therefore, the overall prevalence of low HDL-C was 21.1% (1134/5375).

### 3.3. Gender and Age Difference in Dyslipidemia Prevalence

As shown in Table 1, the prevalence of total dyslipidemia, isolated hypercholesterolemia and low HDL-C in women was significantly higher than in men, whereas no significant difference for isolated hypertriglyceridemia and mixed hyperlipidemia was noted between the genders. 

**Figure 1:**
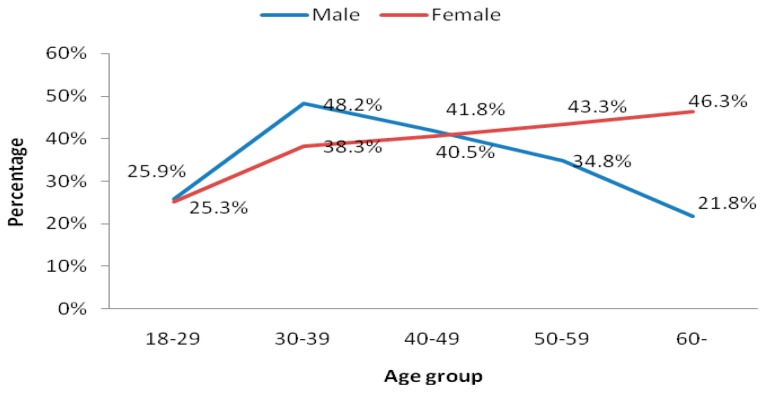
Prevalence of dyslipidemia by different age groups and gender.

As shown in Figure 1, significant differences were found in different age groups (*p <* 0.05), the peak prevalence of dyslipidemia in men was between 30 and 39 years, and then declined gradually; in women, the prevalence of dyslipidemia increased with age (*p <* 0.05), with the peak prevalence occurring after age 60. Dyslipidemia was significantly more prevalent in men under 50 years old than in women, but more prevalent in women 50 and older than men in that age group (*p <* 0.05). 

**Table 1:** The serum lipid concentrations and dyslipidemia prevalence of the study population by different gender.

Characteristics	Total (n = 5375)	Male (n = 2030)	Female (n = 3345)	*p*-Value
TG (mmol/L), geometric mean	1.74	1.74	1.75	0.791
TC (mmol/L), mean (SD)	4.9 ± 1.0	4.9 ± 0.9	5.1 ± 0.1	0.000
HDL_C (mmol/L), mean (SD)	1.5 ± 0.4	1.5 ± 0.5	1.6 ± 0.4	0.000
Dyslipidemia, % (95% CI)	37.4 (36.7, 38.0)	29.1 (28.2, 30.2)	42.4 (41.5, 43.3)	0.000
Isolated hypertriglyceridemia, % (95% CI)	16.5 (16.0, 17.0)	16.3 (15.5, 17.1)	16.6 (16.0, 17.3)	0.762
Isolated hypercholesterolemia, % (95% CI)	5.5 (5.2, 5.8)	3.5 (3.1. 3.9)	6.7 (6.3, 7.1)	0.000
Mixed hyperlipidemia, % (95% CI)	4.9 (4.6, 5.2)	4.2 (3.8, 4.6)	5.4 (5.0, 5.8)	0.067
Isolated low HDL-C, % (95% CI)	10.5 (10.0, 10.9)	5.1 (4.6, 5.6)	13.7 (13.1, 14.3)	0.000
Age-standardized dyslipidemia *, % (95% CI)	35.5 (34.8, 36.1)	34.4 (33.3, 35.4)	37.6 (36.7, 38.4)	0.000

Abbreviations: TG, total hypertriglyceridemia; TC, total cholesterol; HDL-C, high-density lipoprotein cholesterol; SD, standard deviation; CI, confidence interval. * The age-standardized prevalence was calculated based on China’ 2010 census data.

### 3.4. Factors Associated with Dyslipidemia 

On univariate analysis, the percentage of above junior high school education level, habitual drinking and current smoking, BMI, and waist circumference were all significantly higher in the dyslipidemia group than in the control group, but the percentage of male, regular physical activity, and age were lower (Table 2). 

**Table 2:** Anthropometric data in 5375 participants with and without dyslipidemia in Chongqing, China.

Characteristics	All subjects (n = 5375)	Male (n = 2037)	Female (n = 3357)	With Dyslipidemia (n = 2009 )					Without Dyslipidemia (n = 3366)	*p*-Value *
				Overall	Isolated Hypertriglyceridemia (n = 887)	Isolated Hypercholesterolemia (n = 295)	Mixed Hyperlipidemia (n = 265)	Isolated Low HDL-C (n = 562)		
Male, n (%)	2030 (37.8)	−	−	592 (29.5)	331 (37.3)	71 (24.1)	86 (32.5)	104 (18.5)	1438 (42.7)	0.000
Age (years), mean (SD)	57.7 (13.2)	59.7 (13.2)	56.6 (13.1）	57.1 (12.6)	57.0 (12.3)	60.8 (10.6)	58.5 (11.4)	54.7 (14.1)	58.1 (13.5)	0.002
≥Junior high school education	614 (11.4)	298 (14.6)	316 (9.4)	259 (12.8)	127 (14.3)	16 (5.4)	30 (11.3)	86 (15.3)	355 (10.5)	0.009
BMI (kg/m^2^), mean (SD)	23.9 (3.4)	23.4 (3.2)	24.2 (3.4)	25.1 (3.3)	25.5 (3.4)	23.9 (3.1)	25.8 (3.1)	24.9 (3.4)	23.2 (3.1)	0.002
WC (cm), mean (SD)	81.9 (9.4)	82.6 (9.5)	81.5 (9.4)	85.1 (9.5)	86.5 (9.3)	82.0 (9.3)	87.2 (8.8)	85.1 (9.5)	80.0 (8.9)	0.001
Regular physical activity, n (%)	2014 (37.5)	603 (29.1)	1411 (42.0)	607 (30.2)	290 (32.7)	72 (24.4)	60 (22.6)	185 (32.9)	1407 (41.8)	0.002
Habitual drinking, n (%)	4086 (76.0)	1810 (88.8)	2276 (67.8)	1612 (80.2)	675 (76.1)	241 (81.7)	206 (77.7)	490 (87.2)	2474 (73.5)	0.000
Current smoking, n (%)	3141 (58.4)	1752 (86.0)	1389 (41.4)	1202 (59.8)	511 (57.6)	169 (57.3)	147 (55.5)	375（66.7)	1939 (57.6)	0.109

* *p*-value for comparison between subjects with and without dyslipidemia. Abbreviations: BMI, body mass index; WC, waist circumference.

We used multivariable logistic regression examine the associations among age, education level, BMI, waist circumference, physical activity, habitual drinking and current smoking with the odds of dyslipidemia by different gender. The results showed that education level above junior high school, obesity and central obesity were all significantly associated with an increased risk of dyslipidemia for both men and women, whereas regular physical activity was an independent protective factor (Table 3). In addition, older age was associated with an increased risk of dyslipidemia for women, whereas an inverse relationship was observed for men.

**Table 3:** Multivariable-adjusted odds ratios for dyslipidemia by gender.

Variables	Male		Female	
	OR (95% CI)	*p* Value	OR (95% CI)	*p* Value
Age, per 10-year increment	0.82 (0.78, 0.86)	0.000	1.16 (1.13, 1.19)	0.000
≥Junior high school education	1.35 (1.20, 1.50)	0.043	1.26 (1.14, 1.39)	0.046
Regular physical activity	0.79 (0.68, 0.90)	0.032	0.83 (0.76, 0.91)	0.020
Central obesity **^#^**	1.90 (1.76, 2.04)	0.000	1.72 (1.63, 1.80)	0.000
Obesity **^#^**	2.73 (2.61, 2.85)	0.000	1.52 (1.45, 1.59)	0.000

**^#^** References were not central obesity and not obesity. Abbreviations: CI confidence intervals, OR odds ratio.

## 4. Discussion

The increasing prevalence of dyslipidemia has become a worldwide public health problem, and the prevalence varies widely according to the socioeconomic, cultural and ethnic characteristics. This study was the first time to analyze the prevalence of dyslipidemia among Chongqing adults based on NCEP-ATP III criteria. The results revealed that the age-standardized prevalence among Chongqing adults was 35.5%, which was considerably higher than the national reported prevalence of dyslipidemia according to the Chinese National Nutrition and Health Survey in 2002 (18.6%) [18], whereas slightly lower than was found in some cities in China [19,20,21,22]. The differences between this study and other studies may be due to different genetic predisposition, socioeconomic stratum and lifestyles of the studied subjects, as well as diagnostic criteria used.

To allow comparisons with other countries, the NCEP-ATP III criteria rather than the Chinese criteria was used to defined dyslipidemia in our study. The prevalence of dyslipidemia in our study was higher than that in Venezuela [23], Bangladesh [24], and Brazil [25], but lower than that in England [26], and the United States [27]. In our study, the major types of dyslipidemia among Chongqing adults were hypertriglyceridemia and low HDL-C, a finding that agree with those from other studies in Asian countries [23,28,29,30]. This phenomenon probably reflects the increase intake of high simple carbohydrates and high-fat diets in recent decades, which obviously affects the serum triglyceride concentration [31]. Until now, the interventions for low HDL-C were not readily available; therefore, further research into effective intervention measures is needed. 

In our study, the age-adjusted prevalence of dyslipidemia was higher for women (37.6%) than for men (34.4%), which may be related to the differences in the prevalence of overweight, obesity and central obesity. These measurements were significant higher for women than for men. Our study was in conformity with other studies, which also showed the prevalence of dyslipidemia and obesity were more higher for women [32]. However, it was different from that of some other populations in which found a higher percentage of dyslipidemia and obesity for men [21,33,34]. Furthermore, a significant influence of age on lipid levels was observed. The prevalence of dyslipidemia peaking at 30 to 39 years and then declined gradually for men, whereas increasing with age and peaking at 60 years and older for women. Interestingly, the prevalence was higher in men than in women among people under age 50 years, but was reversed among people older than 50 years, which was similar to other study [21]. The highest prevalence of dyslipidemia among 30 to 39 years for men probably due to the intensity of the work pressure coupled with lack of physical activity, which was account for the excessive fat accumulation. This finding indicated that routine screening program for blood lipid levels should be performed and effective interventions programs should be implemented in this age group for men in Chongqing. The increased prevalence of dyslipidemia in older women may be related to the hormonal changes pre- and post- menopausal [35]. 

This study revealed the possible risk factors for dyslipidemia by multivariate analysis. Interestingly, a positive relationship was observed between the level of education and the prevalence of dyslipidemia among the study population. This phenomenon may be related to the better economic level accompanied by over nutrition among people with high education level. Similar to previous studies [23,36,37], obesity and central obesity were identified as risk factors for dyslipidemia in our study. Obesity-associated dyslipidemia has been shown to be atherogenic. Obese individuals have increased atherogenic small, dense LDL particles and elevated levels of apolipoprotein B [38]. Epidemiological studies found that the association between dyslipidemia and abdominal obesity is mediated through an etiopathological mechanism [39]. Therefore, high BMI and WC may be considered as first-stage screening tools to detect dyslipidemic individuals among Chongqing adults.

In addition, it is worth noting that a general inverse relationship was observed between regular physical activity and dyslipidemia. Thus, an appropriate community based prevention strategy emphasizing behavioral changes, especially promoting physical activity, are required to control the epidemic of dyslipidemia.

Some limitations should be noted in this study. First, this was a cross-sectional study; therefore, the causal associations between the risk factors and dyslipidemia cannot be inferred. Second, the investigated population was older than the general adults in Chongqing. Thus, the age-adjusted prevalence of diabetes was calculated. Thirdly, energy intake and genetic factors were not taken into account. Further research involving more potential risk factors is needed. Finally, there are scant data on the frequencies of related disease among study population, therefore, further epidemiological studies are needed to understand more comprehensive information in order to develop prevention and control measures. 

## 5. Conclusions

In summary, dyslipidemia, mainly hypertriglyceridemia and low HDL-C, is very common in Chongqing, China. Gender, age, obesity, central obesity, physical activity and education level are closely related to dyslipidemia. These results highlight the extensive need for routine screening programs for blood lipid levels and appropriate intervention programs aimed at risk factor reduction. 

## Abbreviations

NCEP-ATP III: National Cholesterol Education Program-Adult Treatment Panel III; TC: Total cholesterol; HDL-C: High-density lipoprotein cholesterol; BMI: Body mass index; WC: Waist circumference; SD: standard deviation; CI: confidence interval.
